# Honey gold nanoparticles attenuate the secretion of IL-6 by LPS-activated macrophages

**DOI:** 10.1371/journal.pone.0291076

**Published:** 2023-09-08

**Authors:** John Benjamin W. Duncan, Swarna Basu, Pavithra Vivekanand

**Affiliations:** 1 Department of Biology, Susquehanna University, Selinsgrove, Pennsylvania, United States of America; 2 Department of Chemistry, Susquehanna University, Selinsgrove, Pennsylvania, United States of America; Lady Hardinge Medical College, INDIA

## Abstract

Interleukin-6 (IL-6) is a pleiotropic cytokine that coordinates host immune responses to infection. Though essential to the acute phase response, prolonged IL-6-mediated recruitment of mononuclear cells has been implicated in chronic inflammatory diseases such as rheumatoid arthritis, psoriasis, and Crohn’s disease. Accordingly, identifying novel therapeutics that diminish circulating IL-6 levels could benefit individuals suffering from chronic inflammation. In immunocompetent hosts, bacterial lipopolysaccharide (LPS) recognition by toll-like receptor 4 (TLR4) activates the transcription factor NF-κB, driving macrophage production of IL-6. Interestingly, both citrate-stabilized and ‘green’ synthesized gold nanoparticles (AuNPs) have been shown to modulate the cytokine responses of LPS-activated macrophages. Here we demonstrate that AuNPs, synthesized with commercial and locally sourced honey, downregulate LPS-induced macrophage secretion of IL-6. Compared to LPS-only controls, inhibition of IL-6 levels was observed for all three types of honey AuNPs. The effect was likely driven by honey AuNP-mediated perturbation of the TLR4/NF-κB signaling pathway, as evidenced by a reduction in the phosphorylation of IκB. Further investigation into the anti-inflammatory properties of honey AuNPs may yield novel therapeutics for the treatment of chronic inflammation.

## Introduction

Innate immunity is a powerful first line of defense against infection in which tissue-resident macrophages detect invading pathogens and coordinate effective immune responses. Macrophage cell-surface receptors facilitate pathogen detection via recognition of associated molecular components [1]. These interactions of the pathogen-derived ligands stimulate signaling cascades within macrophages, ultimately inducing the secretion of soluble pro-inflammatory cytokines [2]. In the case of gram-negative bacterial encounter, macrophage toll-like receptor 4 (TLR4) recognizes the lipid A subregion of bacterial lipopolysaccharide (LPS) [3]. LPS-stimulation of TLR4 recruits myeloid differentiation factor 88 (MyD88), thus activating the MyD88-dependent signaling cascade [4]. Upon its association with TLR4, MyD88 recruits interleukin-1 receptor-associated kinase 4, prompting a signaling cascade that culminates in the activation of IκB kinase (IKK). IKK phosphorylates IκB, resulting in the polyubiquitination and degradation of IκB by the proteasome. Since IκB sequesters NF-κB, its degradation enables NF-κB to translocate to the nucleus. There, NF-κB promotes the transcriptional activation of proinflammatory cytokines such as tumor necrosis factor (TNF), interleukin 1 beta (IL-1β), and interleukin 6 (IL-6) [4].

TNF is processed into TNF-α, which promotes inflammation at the site of infection via vasodilation [5]. IL-1β is a critical inflammatory mediator that stimulates macrophage production of other proinflammatory cytokines [6, 7]. IL-6 is a pleiotropic cytokine with paradoxical proinflammatory and anti-inflammatory properties that initiates innate immunity’s acute phase response [8, 9]. Since the dysregulation of each proinflammatory cytokine has been implicated in chronic inflammatory diseases like rheumatoid arthritis and Crohn’s disease, numerous immunomodulatory medications targeting the dysregulated cytokines have been developed [10, 11]. However, modern immunosuppressant drugs are accompanied by a host of deleterious side effects such as increased risk of infection and malignancy [12].

Fortunately, advances in the field of nanobiotechnology have enabled the development of novel therapeutics that circumvent many of these clinical challenges. Due to their small sizes, high surface areas, and relative tunabilities, nanomaterials have emerged as invaluable diagnostic tools, drug-carrying vehicles, and therapeutic agents [13–15]. Within the last two decades, gold nanoparticles (AuNPs) have shown promise in a wide array of biological applications including photothermal cancer therapy [15, 16], bone tissue engineering [17], and antiseptic intervention [18]. As previously summarized by Vines et al., early whole-body photothermal therapies lacked the tissue specificity and penetrative capacity necessary to consistently improve clinical outcomes for cancer patients [16]. Both challenges were overcome using AuNPs, which passively accumulate at the tumor site due to vascular redistribution [19, 20], and can be activated by tissue penetrating near infrared (NIR) light to induce localized hyperthermia [15, 16]. Additionally, surface conjugations of epidermal growth factor receptor antibodies and polyethylene glycol, have enabled AuNPs to target solid tumors and remain undetected by the reticuloendothelial system in circulation, respectively [21, 22]. Finally, morphological modifications to AuNPs have improved their extinction coefficients for more efficient NIR activation in photothermal therapies [23]. Similar translational investigations have shed light onto the relationship between AuNP structure and function both in vitro and in vivo.

It has been well documented that AuNPs accumulate in malignant cell lines and induce cell death to varying degrees depending upon their sizes, shapes, and surface chemistries [24–26]. In 2017, Xie et al. demonstrated that RAW264.7 macrophages utilize shape-dependent endocytic mechanisms for the uptake of gold nanostars, nanotriangles, and nanorods. The team reported that uptake of gold nanotriangles by macrophages was the highest with shape influencing the mode of endocytosis [27]. With regard to surface chemistry, uptake of electropositive AuNPs increased with size, while the reverse was observed for electronegative AuNPs [28]. Furthermore, AuNP coatings readily transform their surface chemistries and thus, dramatically impact their interactions with biological systems [29].

While many researchers utilized knowledge of these phenomena to synthesize more potent anti-cancer AuNPs, Moyano et al. conjugated small compounds to tetraethylene glycol-coated spherical AuNPs to assess their immunomodulatory properties [30]. The AuNP variants (TEGOH, ZDiPen, and ZDiMe), possessed neutral net charges, and averaged 2-nm in diameter. Moyano et al. first tested the AuNPs on J774.2 murine monocytes and RAW264.7 macrophages, reporting an attenuation of TNF-α for cells treated concurrently with TEGOH AuNPs and LPS. In vivo, the team observed a similar decrease in TNF-α levels, also noting that the TEGOH AuNPs remained in circulation for 2 hours until the mice were sacrificed as further evidence of their therapeutic potential [30].

Likely inspired by the success of these murine investigations, Sumbayev et al. probed the immunomodulatory properties of AuNPs in human cell lines. They observed that pretreatment with spherical AuNPs attenuated proinflammatory cytokine responses via the perturbation of IL-1β-dependent processes [31]. Subsequently, ‘green’ AuNPs synthesized with extract derived from *Hypoxis hemerocallidea* were demonstrated to reduce the levels of TNFα and IL-1β produced by LPS stimulated macrophages [32]. Lastly, AuNPs have been successfully synthesized using honey as the reducing agent and were found to exhibit nominal cytotoxicities compared to citrate-stabilized AuNPs [33].

Honey itself has been documented to exhibit anti-oxidant and anti-inflammatory properties attributed to the phenolic acids such as caffeic, ellagic, and ferulic acids, and flavonoids like apigenin, chrysin, and galangin [34, 35]. Phenolic acids have been proposed to work synergistically with flavonoids to produce antioxidant effects that contribute to anti-inflammation [35–37]. Individually, each compound is responsible for biological interactions attributed to immunomodulation. For example, the flavonoid galangin has been demonstrated to inhibit LPS-stimulated rat intestinal epithelial cell production of IL-6, IL-1β, and TNF-α via a reduction of TLR4 and phospho-IκB expression [38]. Since ‘green’ synthetic approaches for AuNPs rely upon the flavonoids and phenolic acids in the natural extracts as reducing, stabilizing, and capping agents [39, 40], we elected to test whether AuNPs, synthesized with locally-sourced and commercial honey, would exhibit similar anti-inflammatory properties on LPS-activated macrophages, derived from a THP-1 human monocytic cell line. Our work demonstrates that AuNPs synthesized using three different types of honey specifically resulted in the inhibition in the levels of IL-6.

## Materials and methods

### Honey AuNP synthesis and characterization

Honey AuNPs were synthesized by modifying a previously developed protocol [33]. Honey variants used in AuNP syntheses include Gunter’s pure clover honey (commercial: CH), locally sourced honey from the fall of 2019 (dark honey: DH), and locally sourced honey from the spring of 2020 (light honey: LH) from Susquehanna University’s apiary. A solution of 1.3 mM tetrachloroauric acid (HAuCl_4_, 10 mL) and of honey (2 g in 10 mL deionized H_2_O) were prepared and placed on heat with vigorous stirring. Once the HAuCl_4_ solution was boiling, the honey solution was added, and the reaction was incubated for 5 minutes under constant heat and stirring until a purple hue was observed. The AuNP solution was removed from heat, covered, and allowed to cool undisturbed. The absorbance spectrum of the 1:10-diluted AuNP solution was measured using a Red Tide USB650 Fiber Optic Spectrometer. Particle size analyses were performed using a Zetasizer Nano ZS (Malvern, PA) and SEM images were obtained using a Verios G4 scanning electron microscope (Thermo-Scientific, Hillsboro, OR) without any additional conductive coating. The AuNP solution was pelleted using a Beckman Coulter Avanti J-26 XP Centrifuge for 30 minutes at 25,000 rpm. The pellet was washed (10 mL of H_2_O) and centrifuged for an additional 15 minutes at 25,000 rpm. FT-IR spectroscopy was carried out using a Thermo Fisher Scientific Nicolet iS50 FT-IR Spectrometer, after allowing the pellet to dry for several days. AuNPs to be used in cell treatments were resuspended in H_2_O after centrifugation at 1/10 the initial volume.

### Cell culture

A THP1 human monocytic cell line was maintained in RPMI 1640 media containing 10% Fetal Bovine Serum (FBS) and 1% penicillin-streptomycin. The line was cultured at 37°C with a 5% CO_2_ saturation.

### Differentiation of THP1 cells

THP1 monocytes were differentiated into macrophages by treatment with phorbol 12-myristate 13-acetate (PMA) at a final concentration of 200 nM for 48 hours. For cytokine response assessments and western blot analyses, the cells were washed twice with RPMI and cultured for an additional 24 hours to improve LPS-responses.

### Cytotoxicity analysis

THP-1 cells were plated in a 96-well plate at a density of 2 x 10^5^ cells/ml and differentiated into macrophages. The cells were washed twice with RPMI and treated with increasing concentrations of each honey AuNP variant in triplicate. After 24 hours of AuNP exposure, cell proliferation and cytotoxicity were assessed using the materials provided and methods detailed in the Cell Proliferation Kit II (XTT) obtained from Sigma-Aldrich.

### Cytokine response assessment

THP-1 cells were plated in a 96-well plate at a density of 2 x 10^5^ cells/ml and differentiated into macrophages. Macrophages were pretreated for one hour with increasing concentrations of honey AuNPs prior to 24 hours of LPS-stimulation (1 μg/ml). Supernatants were collected and relative cytokine concentrations were assessed using the methods detailed in BD Biosciences OptEIA^™^ Human IL-6, IL-1β, and TNF ELISA Kits.

### Western blot analysis

THP-1 cells were plated in a 6-well plate at a density of 1 x 10^6^ cells/ml and differentiated into macrophages. Macrophages were pretreated for two hours with increasing concentrations of commercial honey AuNPs followed by 1 hour of LPS-stimulation (1 μg/ml). Protein extraction was performed with 100 μl of 1x Laemlli buffer with β-mercaptoethanol. The lysates were sonicated for 15 seconds at 25% amplitude and centrifuged for 5 minutes at 14000 rpm at 4°C. Proteins were separated on a 10% SDS-PAGE and transferred to nitrocellulose membranes. The membranes were blocked with 5% non-fat milk in TBST for one hour, washed with TBST, and incubated overnight with anti-phospho-IκB and anti-α-Tubulin antibodies diluted 1:1000 in 5% BSA/TBST (0.1% Tween). The membranes were incubated for one hour at room temperature with goat anti-Mouse-HRP (1:5000) and goat anti-Rabbit-HRP (1:5000) antibodies. Band intensities were quantified with ImageJ software.

### Statistical analyses

All statistical analyses were performed in SPSS. The data represent the mean of three independent trials. Levene’s test of equal variance was performed. If the data failed to meet the condition of equal variance, alpha levels were adjusted from 0.05 to 0.001 to decrease the likelihood of type 1 error. One-way analyses of variance and Student’s t-tests were used to assess differences between honey AuNP treatments of increasing concentrations. Following the determination of no statistical difference between honey AuNP treatments of increasing concentrations, like groups were combined and one-way analyses of variances were performed to elucidate honey AuNP type differences.

## Results and discussion

### AuNP characterization

**Fig 1:**
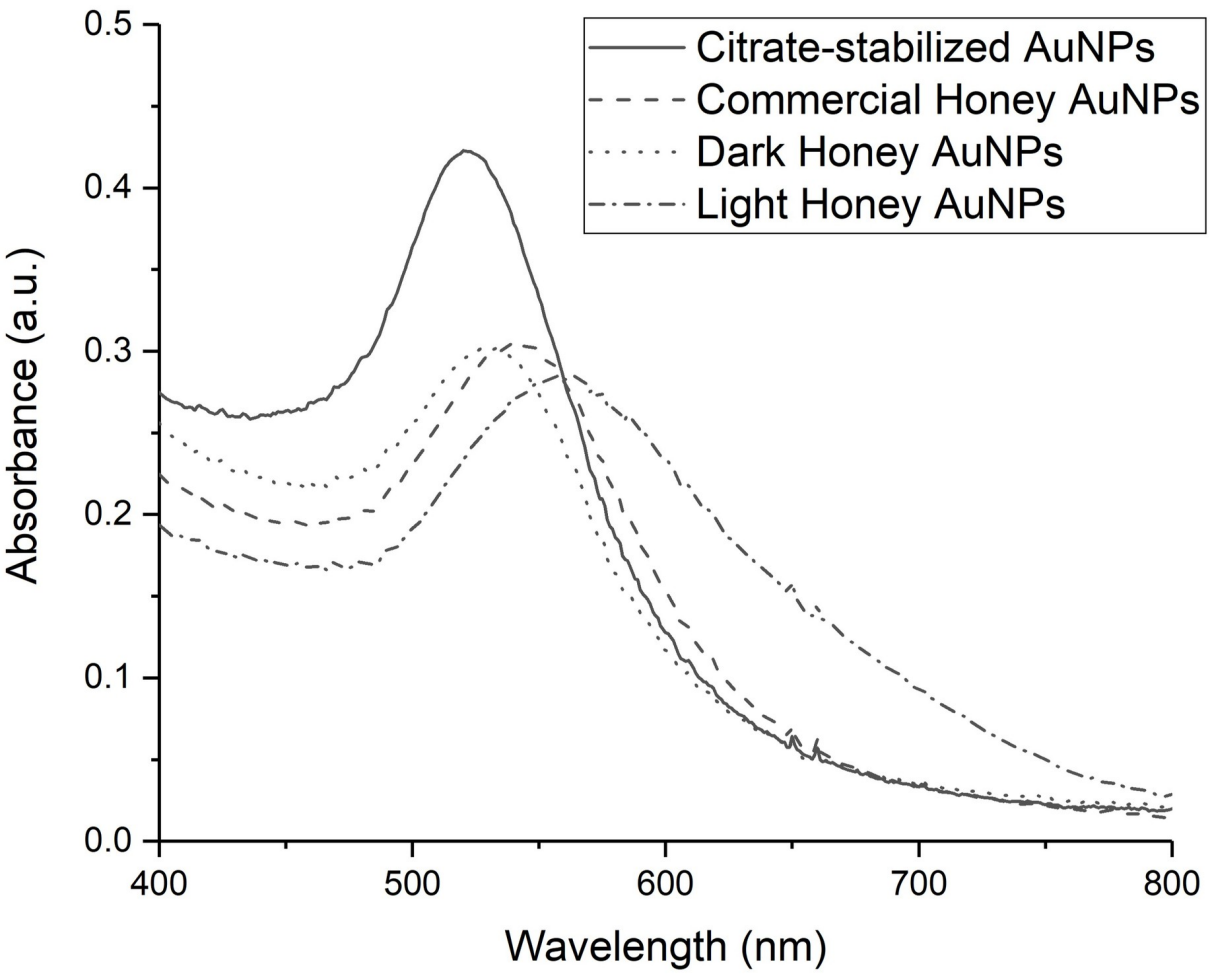
Absorption spectra of the honey mediated AuNP colloids at 22°C. Absorption spectrum of traditional, citrate-stabilized AuNPs has been included for comparison.

‘Green’ synthesized AuNPs were produced with locally sourced and commercial honeys using the methods previously detailed [33]. UV-Vis analyses of the AuNPs (Fig 1) revealed distinct absorption maxima (λ_max_), suggesting morphological differences, attributed to the honey variants used.

**Fig 2:**
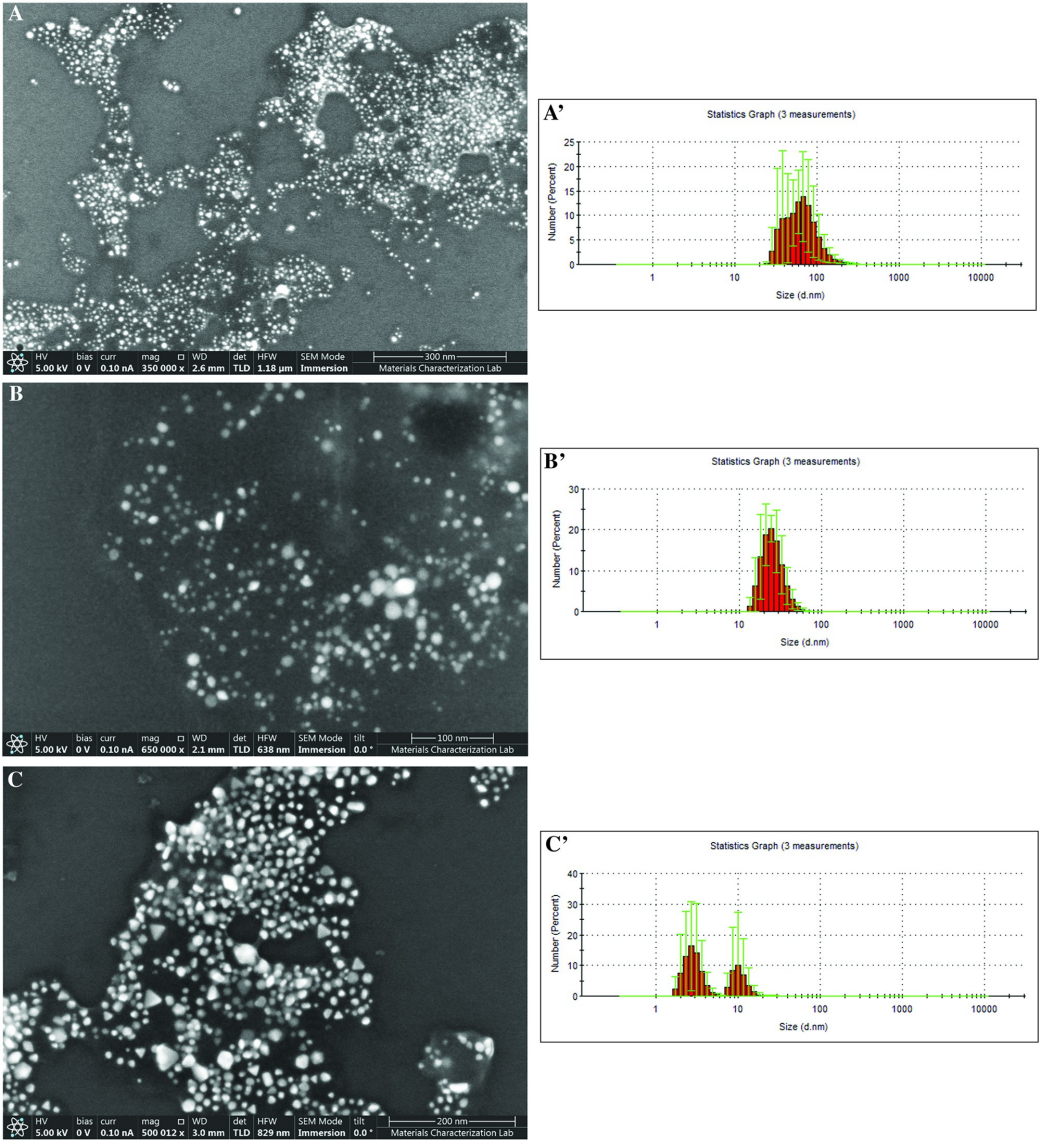
SEM images and particle size distribution of (A, A’) commercial honey AuNP, (B, B’) dark honey AuNP, and (C, C’) light honey AuNP.

Citrate-stabilized AuNPs, produced using the Turkevich method [41], peaked at 527 nm, indicative of the plasmon resonance, whereas commercial (CH AuNP), dark (DH AuNP), and light honey (LH AuNP) AuNPs peaked at 545 nm, 535 nm, and 557 nm, respectively. The broadness of λ_max_ suggested shape and size distinctions within each colloidal AuNP suspension [42]. These distinctions were elucidated via SEM imaging and dynamic light scattering (DLS) particle size analyses (Fig 2). Honey AuNPs ranged from quasi-spherical to triangular in shape. The average particle sizes of the commercial, dark, and light honey AuNPs were 67.9 nm, 25.9 nm, and 5.4 nm, respectively (Fig 2).

The bimodal distribution of DLS reads for the colloidal suspension of light honey AuNPs (Fig 2C’) may be attributed to the presence of both small, quasi-spherical and large, triangular particles therein (Fig 2C). As noted by Khandanlou et al., these shape and size differences can be attributed to flavonoids and phenolic acids in honey, which differentially reduce and cap AuNPs [43]. Depending upon the seasonal availability of flora, bees produce honeys of varying compositions. For this reason, local honey acquired in the fall possessed a dramatically darker hue than the spring harvest.

To assess the functional groups attributed to the various honey AuNP surface conjugates, FT-IR analyses were performed. The FT-IR spectra of citrate-stabilized and honey AuNPs were consistent with the literature [33]. Citrate stabilized AuNPs (S1A Fig) exhibited a broad stretching region from 3100 cm^-1^ to 3750 cm^-1^ due to water’s interaction with free carboxylic acid groups. These groups were denoted by a broad peak around 1618 cm^-1^, while COO^-^ groups were represented by a peak around 1398 cm^-1^. In contrast, the honey AuNP’s FT-IR spectrum revealed a broad, elongated band from 3000–3700 cm^-1^, denoting OH groups associated with sugars in the honey (S1B–S1D Fig). A double peak associated with cellulose and lipids in the honey can be observed around 2934 cm^-1^ and 2883 cm^-1^, while carbonyl stretching is represented as a band around 1642 cm^-1^. The intense peak near 1057 cm^-1^ suggests that proteins from the honey are bound to the AuNPs through free amine groups [33]. The lack of distinction between honey AuNP FT-IR spectra suggest conserved interactions between ionic gold and honey constituents during honey AuNP synthesis.

### Honey AuNPs are not cytotoxic to THP-1 derived macrophages

**Fig 3:**
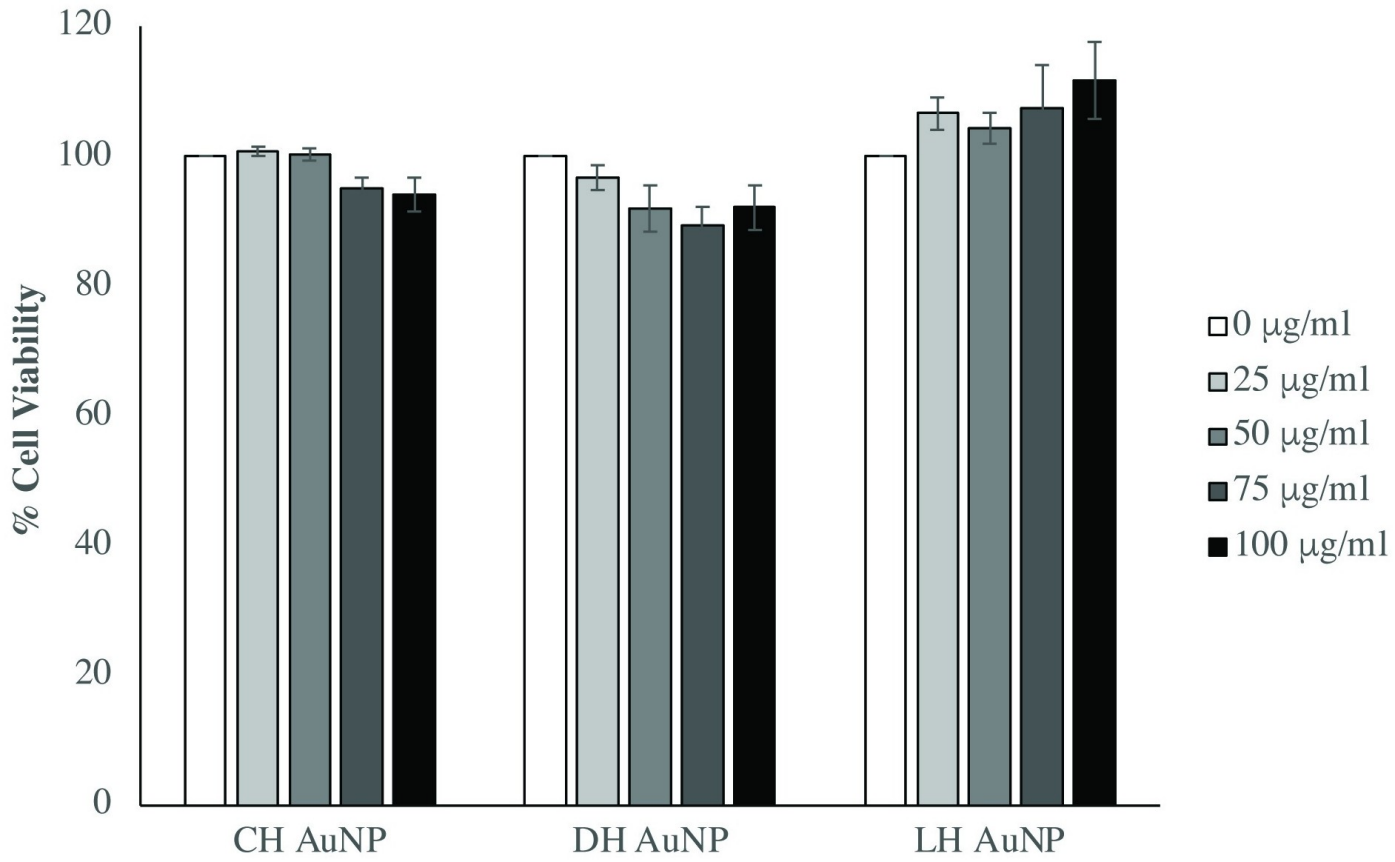
Cell viability is not affected by honey AuNP treatment. Honey AuNPs exhibited nominal cytotoxicities on THP-1-derived macrophages at concentrations up to 100 μg/mL (p > 0.001). Combined results are reflective of three independent assays involving 24-hour exposure of macrophages to increasing concentrations of the honey mediated AuNP variants. Error bars represent +/- SE.

Since AuNPs can induce mitochondrial oxidative stress, cell cycle arrest, and apoptosis [24, 44, 45], an XTT assay was performed to examine whether honey AuNPs could affect the viability of THP-1 derived macrophages. Despite apparent similarities between honey AuNP surface conjugates, morphological distinctions between honey AuNPs necessitated independent cytotoxicity testing. THP-1 cells exposed to 25–100 μg/mL of CH, DH, and LH AuNPs for 24 hours exhibited minimal effects on viability (Fig 3).

### Honey AuNPs inhibit IL-6 secretion

**Fig 4:**
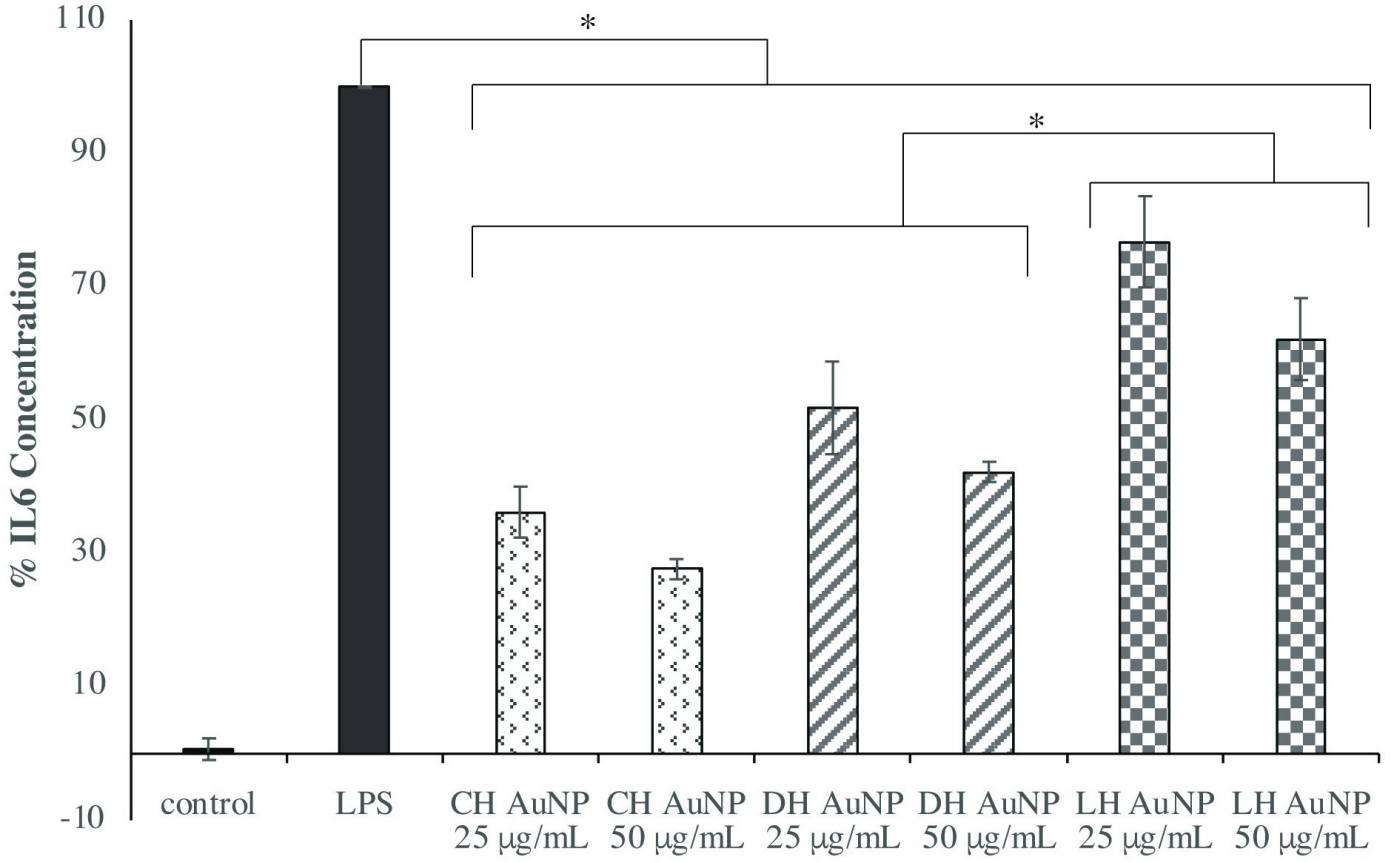
Honey AuNP pretreatment attenuated macrophage secretion of IL-6 (p < 0.001) differentially, depending upon honey AuNP type, but not concentration. Commercial (CH) and dark honey (DH) AuNP treatments lowered macrophage secretion of IL-6 more than light honey (LH) AuNP treatments (p < 0.001). Differences in attenuation between commercial and dark honey AuNP treatments approached significance (p = 0.057). Combined results reflect the mean of three independent ELISAs in which THP-1-derived macrophages were pretreated with honey AuNPs 1 hour prior to LPS stimulation (1 μg/mL) for 24 hours. Error bars represent +/- SE.

To assess the extent to which honey AuNP-pretreatment attenuates macrophage production of various proinflammatory cytokines, ELISAs were performed to examine IL-6, TNFα, and IL-1β levels. Cells were exposed to either 25 or 50 μg/mL of CH, DH, or LH AuNPs prior to stimulation with LPS to activate the TLR4 signaling pathway. Interestingly, the type of honey AuNP had a significant effect on the extent to which macrophage secretion of IL-6 was attenuated (p < 0.001). Compared to the LPS-only control, pretreatment with 50 μg/ml of LH AuNP reduced IL-6 levels to 62%, while pretreatment with 50 μg/ml of CH and DH AuNP reduced IL-6 secretion to 28% and 42%, respectively (p < 0.001) (Fig 4). This observation may be attributed to the flavonoids and phenolic acids within commercial, dark, and light honeys, respectively. Given the uniformity of honey AuNP FT-IR spectra, immunomodulatory differences may be linked to the flavonoids and phenolic acids that contribute to morphological differences between AuNPs, rather than AuNP surface conjugates.

**Fig 5:**
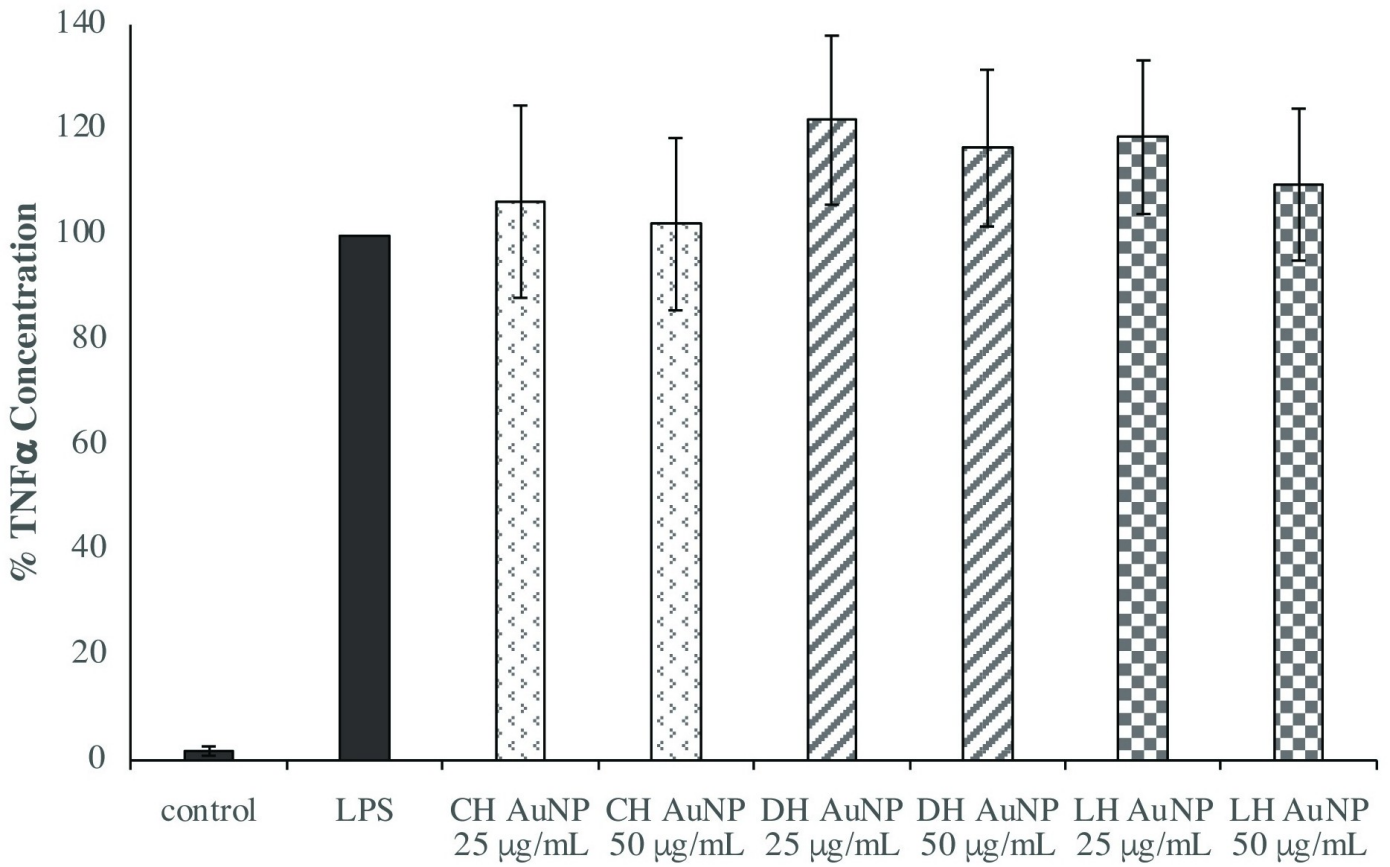
Honey AuNP pretreatment had no effect on macrophage secretion of TNF-α compared to the LPS-only control. Combined results reflect the mean of three independent ELISAs in which THP-1-derived macrophages were pretreated with honey AuNPs 1 hour prior to LPS stimulation (1 μg/mL) for 24 hours. Error bars represent +/- SE.

**Fig 6:**
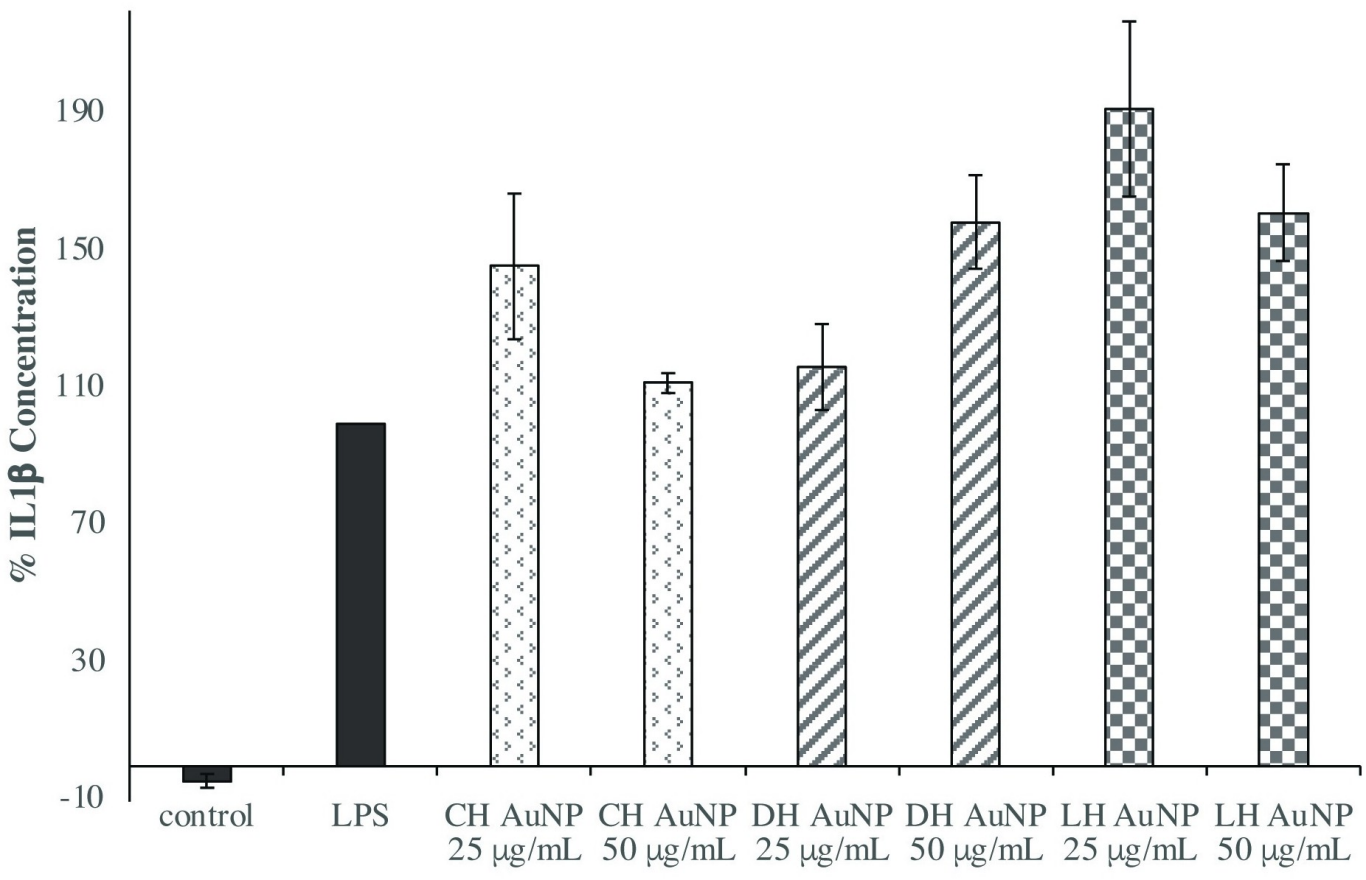
Light honey AuNP pretreatment increased macrophage secretion of IL-1β compared to the LPS-only control (p = 0.01), while the other honey AuNP variants had no effect. Combined results reflect the mean of three independent ELISAs in which THP-1-derived macrophages were pretreated with honey AuNPs 1 hour prior to LPS stimulation (1 μg/mL) for 24 hours. Error bars represent +/- SE.

We subsequently examined the efficacy of honey AuNPs to reduce TNF-α and IL-1β production to determine the effect of gold nanoparticles on different pro-inflammatory cytokines. With the exception of light honey AuNP pretreatment slightly increasing macrophage secretion of IL-1β (Fig 6), commercial and dark honey AuNPs did not have an effect on the levels of either TNF-α or IL-1β (Figs 5 and 6).

The increase in cell secretion of IL-1β for cells pretreated with light honey AuNPs might be a result of NLRP3 inflammasome activation [46]. Zhu et al. reported that gold nanoparticles less than 10 nm in diameter activated the NLRP3 inflammasome, while larger AuNPs had no effect. The average size of the light honey AuNPs was 5.4 nm, while that of commercial and dark honey AuNPs were 67.9 nm and 25.9 nm, respectively, which might explain why only light honey AuNP pretreatment led to an increase in IL-1β levels. Our results are consistent with recent published findings, which demonstrated specific reductions of proinflammatory cytokines in accordance with AuNP size, shape, and surface chemistry. Sumbayev et al. established that pretreatment with small-diameter (5 nm), citrate-stabilized AuNPs reduced TNF-α secretion by IL-1β-stimulated THP-1 cells [31]. Similarly, Elbagory et al. showed that quasi-spherical AuNPs, synthesized with *Hypoxis hemerocallidea* extract, reduced LPS-activated macrophage secretion of IL-1β and TNF-α, but not IL-6 [32].

**Fig 7:**
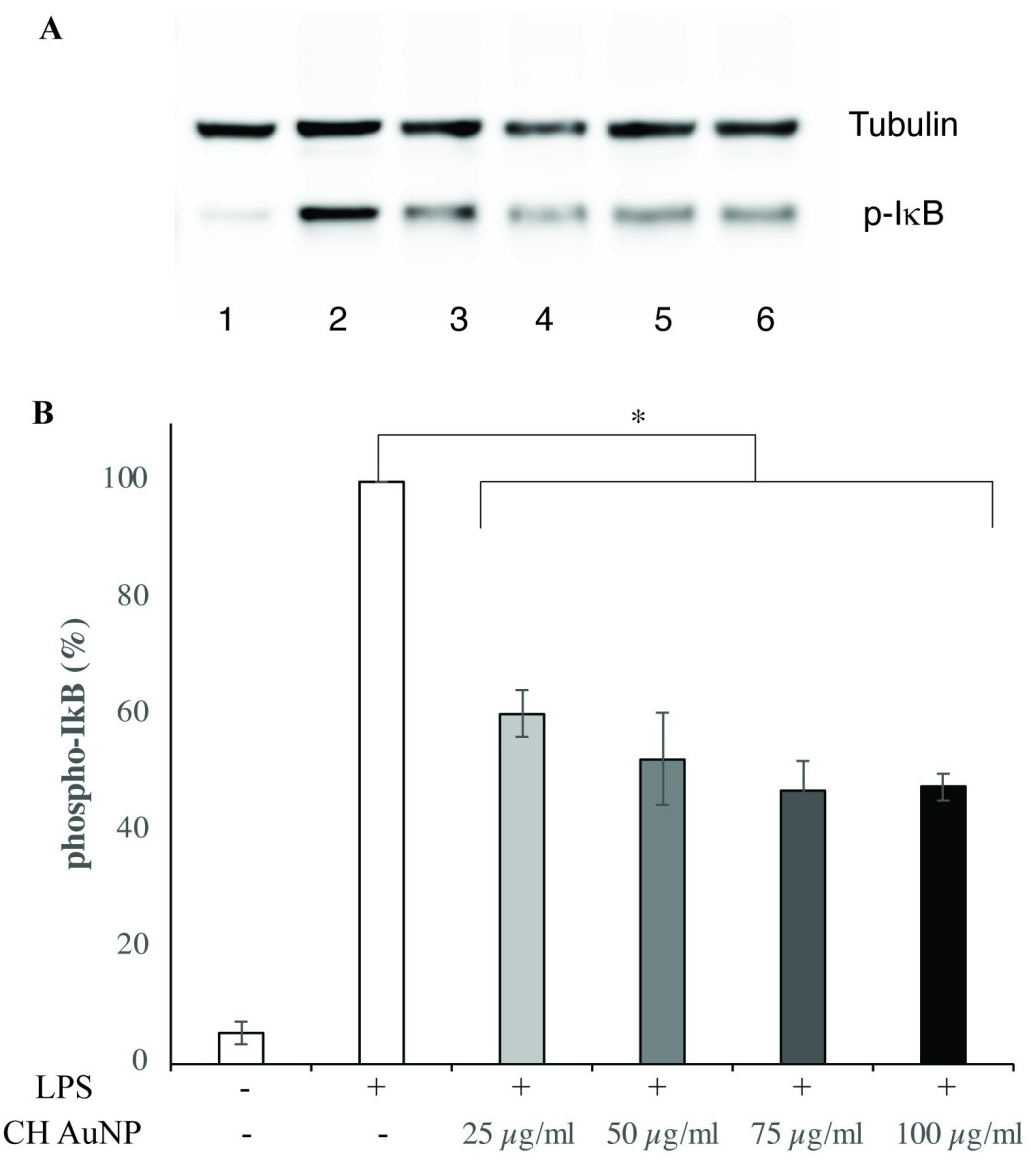
Pretreatment with commercial honey AuNPs decreased levels of phospho-IκB compared to the LPS-only control (p < 0.001). (A) Lane 1 and 2 correspond to lysates from control and LPS treated cells, respectively, while lanes 3–6 correspond to cells treated with LPS and increasing concentrations of CH AuNP (25–100 μg/ml). α-Tubulin was used as a loading control. (B) Quantification of phospho-IκB levels relative to Tubulin. Band intensities were quantified using ImageJ software and converted to percentages with respect to the LPS-only control, and reflects the average of 3 independent experiment. Error bars represent +/- SE.

To investigate the signaling pathway implicated in the honey AuNP-mediated attenuation of IL-6, western blots were performed to examine the levels of phosphorylated IκB (pIκB). Cells were pretreated for 2 hours with commercial honey AuNPs and then stimulated for 1 hour with LPS (1 μg/mL) prior to lysis. Honey AuNP pretreatment resulted in a 40–53% decrease in the amount of pIκB compared to the LPS-only control (Fig 7, and S2 Fig). As unphosphorylated IκB sequesters NFκB in the cytoplasm, the honey AuNP-mediated attenuation of IκB phosphorylation suggests a reduced activation of NFκB, which would explain the decrease in macrophage secretion of IL-6. This may also implicate the flavonoid galangin, which inhibits NFκB while simultaneously reducing macrophage expression of TLR4 and pIκB [38]. Galangin independently prevents NFκB activation by occupying TLR4’s binding site without stimulating the receptor. However, the reduction in TLR4 and pIκB expression is mediated by an upregulation of the anti-inflammatory cytokines interleukin-10 and transforming growth factor-β [38].

The reduction in secreted IL-6, but not IL-1β and TNF-α could be attributed to NFκB binding differentially to associated promoter regions. Enhanced NFκB binding to IL-1β and TNF-α-associated regions would explain why similar levels of the IL-1β and TNF-α were produced in light of honey AuNP-mediated reduction in NFκB activation.

Given that elevated IL-6 levels are implicated in a host of chronic inflammatory diseases, the capacity of honey AuNPs to attenuate macrophage secretion of IL-6 may present a novel therapeutic option for affected individuals. Since AuNPs are highly tunable, targeting molecules could be affixed to their surface for the site-specific treatment of inflammation. Developing potent, targeted anti-inflammatories would overcome one of the major clinical challenges of current anti-inflammatory medications, which act on the whole body and reduce patient immunocompetencies [12].

## Conclusion

We have demonstrated that AuNPs synthesized using commercial and locally-sourced honeys exhibit immunomodulatory properties. Pretreating THP-1-derived macrophages with honey AuNPs before LPS stimulation revealed a specific attenuation of IL-6 production. This observation suggests that some of honey’s potent anti-inflammatory properties are conserved during AuNP synthesis. Mechanistic investigations into the attenuation of IL-6 revealed decreased intracellular macrophage levels of pIκB, a constituent of the TLR4/NFκB pathway. Further investigations of the honey AuNP surface conjugates and their immunomodulatory mechanisms of action could be examined by performing an RNA-seq analysis. This may enable the development of potent, cytokine-specific, anti-inflammatory AuNPs. Given the therapeutic relevance of gold nanoparticles, honey AuNPs may emerge as novel treatment options for chronic inflammatory diseases such as rheumatoid arthritis or Crohn’s disease.

## Supporting information

S1 Fig FT-IR spectra of honey gold nanoparticles (A) citrate AuNPs, (B) commercial honey AuNPs, (C) Dark honey AuNPs, (D) light honey AuNPs. (TIF)

S2 Fig Raw data of western blotting to detect relative levels of phospho-IκB in cells treated with AuNP compared to control cells. Three independent western blots were performed and the average was used to quantify the levels of phospho-IκB shown in Fig 7B.
(TIF)

S1 Data (TIF)

S2 Data (TIF)

## References

[1] Beutler B. Innate immunity: an overview. Mol Immunol. 2004;40(12):845–59. doi: 10.1016/j.molimm.2003.10.005 . 14698223

[2] Netea MG, Latz E, Mills KH, O’Neill LA. Innate immune memory: a paradigm shift in understanding host defense. Nat Immunol. 2015;16(7):675–9. doi: 10.1038/ni.3178 . 26086132

[3] Maeshima N, Fernandez RC. Recognition of lipid A variants by the TLR4-MD-2 receptor complex. Front Cell Infect Microbiol. 2013;3:3. Epub 20130212. doi: 10.3389/fcimb.2013.00003 . 23408095 PMC3569842

[4] Kawai T, Akira S. TLR signaling. Cell Death Differ. 2006;13(5):816–25. doi: 10.1038/sj.cdd.4401850 . 16410796

[5] Bradley JR. TNF-mediated inflammatory disease. J Pathol. 2008;214(2):149–60. doi: 10.1002/path.2287 . 18161752

[6] Kaneko N, Kurata M, Yamamoto T, Morikawa S, Masumoto J. The role of interleukin-1 in general pathology. Inflamm Regen. 2019;39:12. Epub 20190606. doi: 10.1186/s41232-019-0101-5 . 31182982 PMC6551897

[7] Ren K, Torres R. Role of interleukin-1beta during pain and inflammation. Brain Res Rev. 2009;60(1):57–64. Epub 20081231. doi: 10.1016/j.brainresrev.2008.12.020 . 19166877 PMC3076185

[8] Hirano T. IL-6 in inflammation, autoimmunity and cancer. Int Immunol. 2021;33(3):127–48. doi: 10.1093/intimm/dxaa078 . 33337480 PMC7799025

[9] Tanaka T, Narazaki M, Kishimoto T. IL-6 in inflammation, immunity, and disease. Cold Spring Harb Perspect Biol. 2014;6(10):a016295. Epub 20140904. doi: 10.1101/cshperspect.a016295 . 25190079 PMC4176007

[10] McInnes IB, Schett G. Cytokines in the pathogenesis of rheumatoid arthritis. Nat Rev Immunol. 2007;7(6):429–42. doi: 10.1038/nri2094 . 17525752

[11] Strober W, Zhang F, Kitani A, Fuss I, Fichtner-Feigl S. Proinflammatory cytokines underlying the inflammation of Crohn’s disease. Curr Opin Gastroenterol. 2010;26(4):310–7. doi: 10.1097/MOG.0b013e328339d099 . 20473158 PMC3681421

[12] Bamoulid J, Staeck O, Halleck F, Khadzhynov D, Brakemeier S, Durr M, et al. The need for minimization strategies: current problems of immunosuppression. Transpl Int. 2015;28(8):891–900. Epub 20150318. doi: 10.1111/tri.12553 . 25752992

[13] Sztandera K, Gorzkiewicz M, Klajnert-Maculewicz B. Gold Nanoparticles in Cancer Treatment. Mol Pharm. 2019;16(1):1–23. Epub 20181130. doi: 10.1021/acs.molpharmaceut.8b00810 . 30452861

[14] Daraee H, Eatemadi A, Abbasi E, Fekri Aval S, Kouhi M, Akbarzadeh A. Application of gold nanoparticles in biomedical and drug delivery. Artif Cells Nanomed Biotechnol. 2016;44(1):410–22. Epub 20140917. doi: 10.3109/21691401.2014.955107 . 25229833

[15] Hu X, Zhang Y, Ding T, Liu J, Zhao H. Multifunctional Gold Nanoparticles: A Novel Nanomaterial for Various Medical Applications and Biological Activities. Front Bioeng Biotechnol. 2020;8:990. Epub 20200813. doi: 10.3389/fbioe.2020.00990 . 32903562 PMC7438450

[16] Vines JB, Yoon JH, Ryu NE, Lim DJ, Park H. Gold Nanoparticles for Photothermal Cancer Therapy. Front Chem. 2019;7:167. Epub 20190405. doi: 10.3389/fchem.2019.00167 . 31024882 PMC6460051

[17] Vial S, Reis RL, Oliveira JM. Recent advances using gold nanoparticles as a promising multimodal tool for tissue engineering and regenerative medicine. Current Opinion in Solid State and Materials Science. 2017;21(2):92–112. doi: 10.1016/j.cossms.2016.03.006

[18] Tao C. Antimicrobial activity and toxicity of gold nanoparticles: research progress, challenges and prospects. Lett Appl Microbiol. 2018;67(6):537–43. Epub 20181025. doi: 10.1111/lam.13082 . 30269338

[19] Goddard ZR, Marin MJ, Russell DA, Searcey M. Active targeting of gold nanoparticles as cancer therapeutics. Chem Soc Rev. 2020;49(23):8774–89. Epub 20201022. doi: 10.1039/d0cs01121e . 33089858

[20] Jain S, Hirst DG, O’Sullivan JM. Gold nanoparticles as novel agents for cancer therapy. Br J Radiol. 2012;85(1010):101–13. Epub 20111018. doi: 10.1259/bjr/59448833 . 22010024 PMC3473940

[21] Li SD, Huang L. Stealth nanoparticles: high density but sheddable PEG is a key for tumor targeting. J Control Release. 2010;145(3):178–81. Epub 20100323. doi: 10.1016/j.jconrel.2010.03.016 . 20338200 PMC2902652

[22] Song L, Falzone N, Vallis KA. EGF-coated gold nanoparticles provide an efficient nano-scale delivery system for the molecular radiotherapy of EGFR-positive cancer. Int J Radiat Biol. 2016;92(11):716–23. Epub 20160321. doi: 10.3109/09553002.2016.1145360 . 26999580 PMC5116916

[23] Chen H, Kou X, Yang Z, Ni W, Wang J. Shape- and size-dependent refractive index sensitivity of gold nanoparticles. Langmuir. 2008;24(10):5233–7. Epub 20080425. doi: 10.1021/la800305j . 18435552

[24] Favi PM, Valencia MM, Elliott PR, Restrepo A, Gao M, Huang H, et al. Shape and surface chemistry effects on the cytotoxicity and cellular uptake of metallic nanorods and nanospheres. J Biomed Mater Res A. 2015;103(12):3940–55. Epub 20150921. doi: 10.1002/jbm.a.35518 . 26053238

[25] Wozniak A, Malankowska A, Nowaczyk G, Grzeskowiak BF, Tusnio K, Slomski R, et al. Size and shape-dependent cytotoxicity profile of gold nanoparticles for biomedical applications. J Mater Sci Mater Med. 2017;28(6):92. Epub 2017/05/13. doi: 10.1007/s10856-017-5902-y . 28497362

[26] Dickson J, Weaver B, Vivekanand P, Basu S. Anti-neoplastic Effects of Gold Nanoparticles Synthesized Using Green Sources on Cervical and Melanoma Cancer Cell Lines. BioNanoScience. 2023;13(1):194–202. doi: 10.1007/s12668-022-01056-z

[27] Xie X, Liao J, Shao X, Li Q, Lin Y. The Effect of shape on Cellular Uptake of Gold Nanoparticles in the forms of Stars, Rods, and Triangles. Sci Rep. 2017;7(1):3827. Epub 20170619. doi: 10.1038/s41598-017-04229-z . 28630477 PMC5476625

[28] Jiang Y, Huo S, Mizuhara T, Das R, Lee YW, Hou S, et al. The Interplay of Size and Surface Functionality on the Cellular Uptake of Sub-10 nm Gold Nanoparticles. ACS Nano. 2015;9(10):9986–93. Epub 20151007. doi: 10.1021/acsnano.5b03521 . 26435075 PMC5848075

[29] Zhang J, Mou L, Jiang X. Surface chemistry of gold nanoparticles for health-related applications. Chem Sci. 2020;11(4):923–36. Epub 20200115. doi: 10.1039/c9sc06497d . 34084347 PMC8145530

[30] Moyano DF, Liu Y, Ayaz F, Hou S, Puangploy P, Duncan B, et al. Immunomodulatory effects of coated gold nanoparticles in LPS-stimulated in vitro and in vivo murine model systems. Chem. 2016;1(2):320–7. Epub 20160811. doi: 10.1016/j.chempr.2016.07.007 . 28255579 PMC5328597

[31] Sumbayev VV, Yasinska IM, Garcia CP, Gilliland D, Lall GS, Gibbs BF, et al. Gold nanoparticles downregulate interleukin-1beta-induced pro-inflammatory responses. Small. 2013;9(3):472–7. Epub 20121030. doi: 10.1002/smll.201201528 . 23112137

[32] Elbagory AM, Hussein AA, Meyer M. The In Vitro Immunomodulatory Effects Of Gold Nanoparticles Synthesized From Hypoxis hemerocallidea Aqueous Extract And Hypoxoside On Macrophage And Natural Killer Cells. Int J Nanomedicine. 2019;14:9007–18. Epub 20191119. doi: 10.2147/IJN.S216972 . 31819415 PMC6875510

[33] Boldeiu A, Simion M, Mihalache I, Radoi A, Banu M, Varasteanu P, et al. Comparative analysis of honey and citrate stabilized gold nanoparticles: In vitro interaction with proteins and toxicity studies. J Photochem Photobiol B. 2019;197:111519. Epub 20190605. doi: 10.1016/j.jphotobiol.2019.111519 . 31228688

[34] Navaei-Alipour N, Mastali M, Ferns GA, Saberi-Karimian M, Ghayour-Mobarhan M. The effects of honey on pro- and anti-inflammatory cytokines: A narrative review. Phytother Res. 2021;35(7):3690–701. Epub 20210309. doi: 10.1002/ptr.7066 . 33751689

[35] Vallianou NG, Gounari P, Skourtis A, Panagos J, Kazazis C. Honey and its Anti-Inflammatory, Anti-Bacterial and Anti-Oxidant Properties. General Medicine. 2014. doi: 10.4172/2327-5146.1000132

[36] Subramanian AP, John AA, Vellayappan MV, Balaji A, Jaganathan SK, Mandal M, et al. Honey and its Phytochemicals: Plausible Agents in Combating Colon Cancer through its Diversified Actions. Journal of Food Biochemistry. 2016;40(4):613–29. doi: 10.1111/jfbc.12239

[37] Zhang L, Virgous C, Si H. Synergistic anti-inflammatory effects and mechanisms of combined phytochemicals. J Nutr Biochem. 2019;69:19–30. Epub 20190331. doi: 10.1016/j.jnutbio.2019.03.009 . 31048206

[38] Cai SQ, Zhang Q, Zhao XH, Shi J. The In Vitro Anti-Inflammatory Activities of Galangin and Quercetin towards the LPS-Injured Rat Intestinal Epithelial (IEC-6) Cells as Affected by Heat Treatment. Molecules. 2021;26(24). Epub 20211210. doi: 10.3390/molecules26247495 . 34946578 PMC8703769

[39] Bharadwaj KK, Rabha B, Pati S, Sarkar T, Choudhury BK, Barman A, et al. Green Synthesis of Gold Nanoparticles Using Plant Extracts as Beneficial Prospect for Cancer Theranostics. Molecules. 2021;26(21). Epub 20211022. doi: 10.3390/molecules26216389 . 34770796 PMC8586976

[40] Jannathul Firdhouse M, Lalitha P. Biogenic green synthesis of gold nanoparticles and their applications—A review of promising properties. Inorganic Chemistry Communications. 2022;143:109800. doi: 10.1016/j.inoche.2022.109800

[41] Turkevich J, Stevenson PC, Hillier J. A study of the nucleation and growth processes in the synthesis of colloidal gold. Discussions of the Faraday Society. 1951;11(0):55–75. doi: 10.1039/DF9511100055

[42] Saion E, Gharibshahi E, Naghavi K. Size-controlled and optical properties of monodispersed silver nanoparticles synthesized by the radiolytic reduction method. Int J Mol Sci. 2013;14(4):7880–96. Epub 20130411. doi: 10.3390/ijms14047880 . 23579953 PMC3645722

[43] Khandanlou R, Murthy V, Wang H. Gold nanoparticle-assisted enhancement in bioactive properties of Australian native plant extracts, Tasmannia lanceolata and Backhousia citriodora. Mater Sci Eng C Mater Biol Appl. 2020;112:110922. Epub 20200404. doi: 10.1016/j.msec.2020.110922 . 32409072

[44] Kowsalya E, MosaChristas K, Jaquline CRI, Balashanmugam P, Devasena T. Gold nanoparticles induced apoptosis via oxidative stress and mitochondrial dysfunctions in MCF-7 breast cancer cells. Applied Organometallic Chemistry. 2021;35(1):e6071. doi: 10.1002/aoc.6071

[45] Ramalingam V, Revathidevi S, Shanmuganayagam T, Muthulakshmi L, Rajaram R. Biogenic gold nanoparticles induce cell cycle arrest through oxidative stress and sensitize mitochondrial membranes in A549 lung cancer cells. RSC Advances. 2016;6(25):20598–608. doi: 10.1039/C5RA26781A

[46] Zhu M, Du L, Zhao R, Wang HY, Zhao Y, Nie G, et al. Cell-Penetrating Nanoparticles Activate the Inflammasome to Enhance Antibody Production by Targeting Microtubule-Associated Protein 1-Light Chain 3 for Degradation. ACS Nano. 2020;14(3):3703–17. Epub 20200220. doi: 10.1021/acsnano.0c00962 . 32057231 PMC7457719

