# Supplementary figures and images for: Honey gold nanoparticles attenuate the secretion of IL-6 by LPS-activated macrophages
Source: PLoS One. 2023 Sep 8;18(9):e0291076. doi: 10.1371/journal.pone.0291076 (PMC10490926; doi:10.1371/journal.pone.0291076)

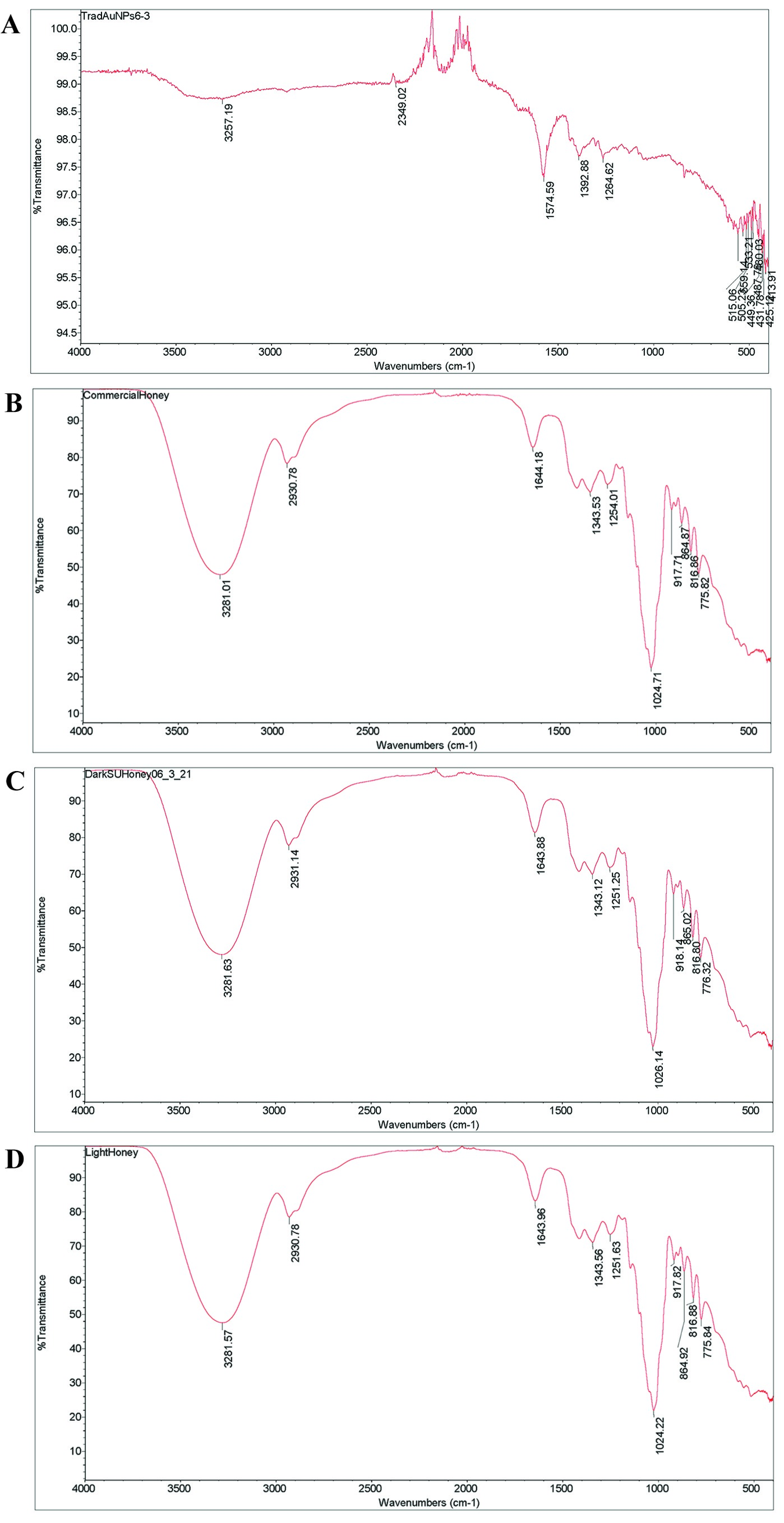

Supplement: S1 Fig — (TIF) [file pone.0291076.s001.tif]

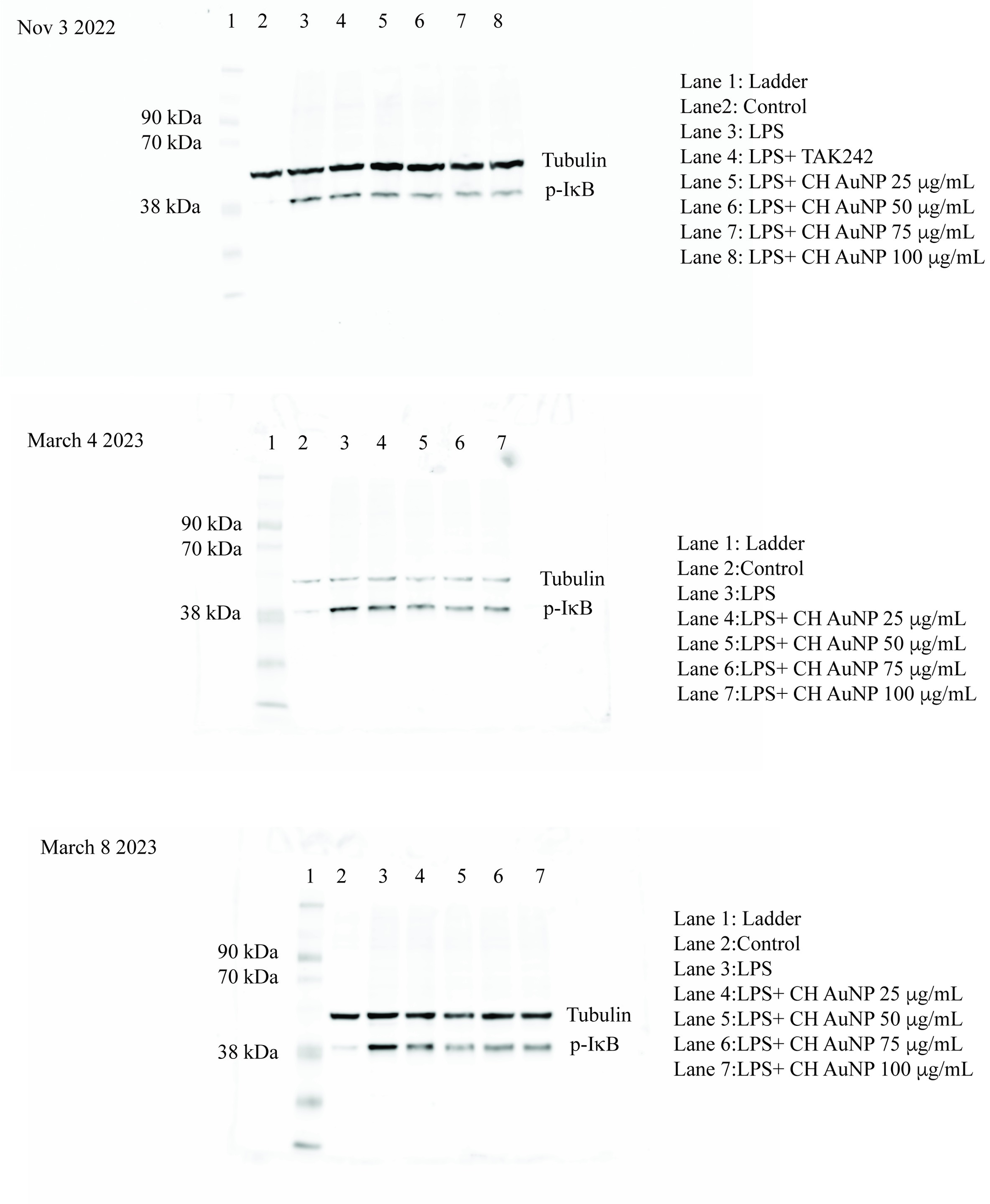

Supplement: S2 Fig — Three independent western blots were performed and the average was used to quantify the levels of phospho-IκB shown in Fig 7B. (TIF) [file pone.0291076.s002.tif]

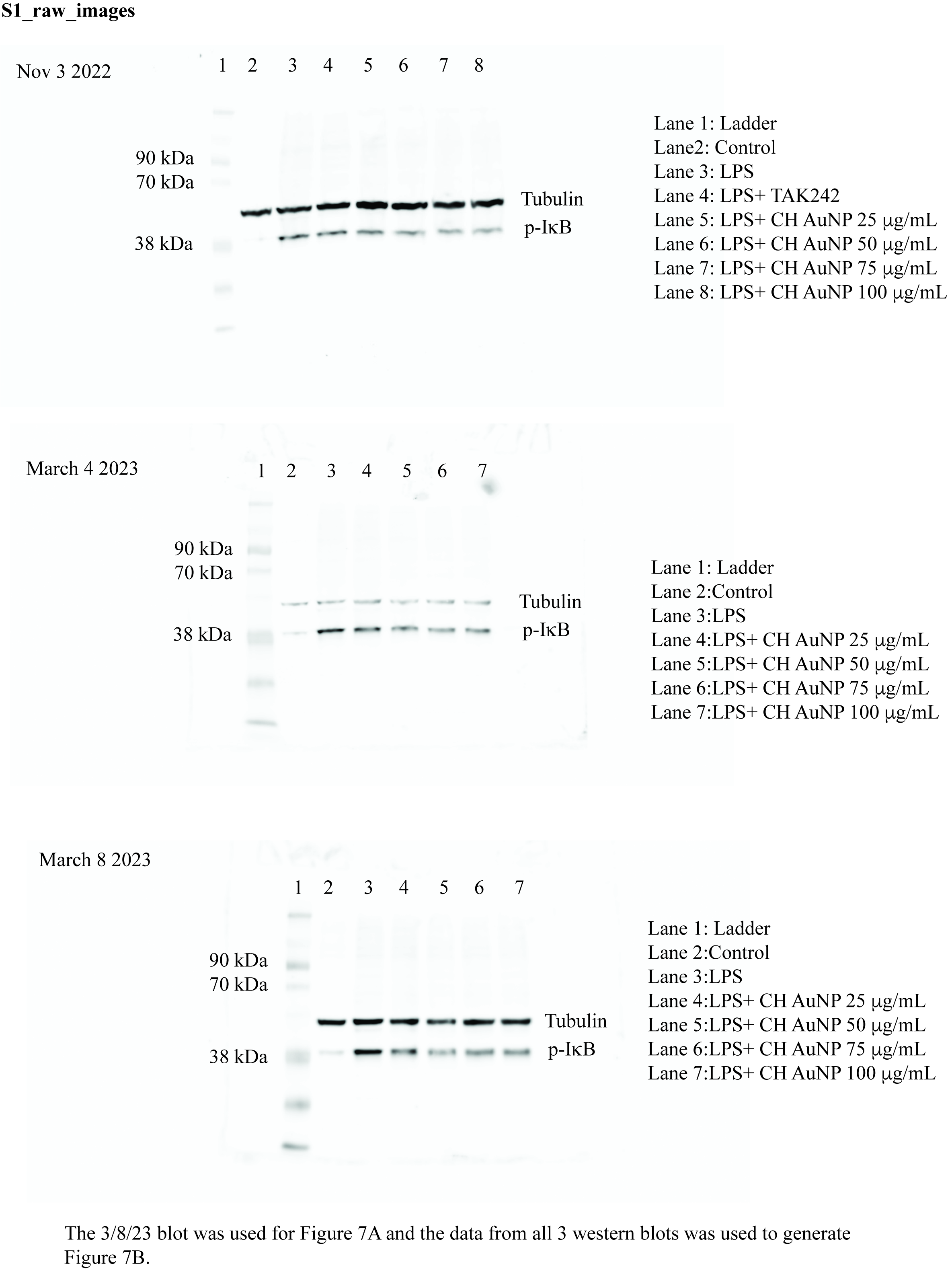

Supplement: S1 Data — (TIF) [file pone.0291076.s003.tif]

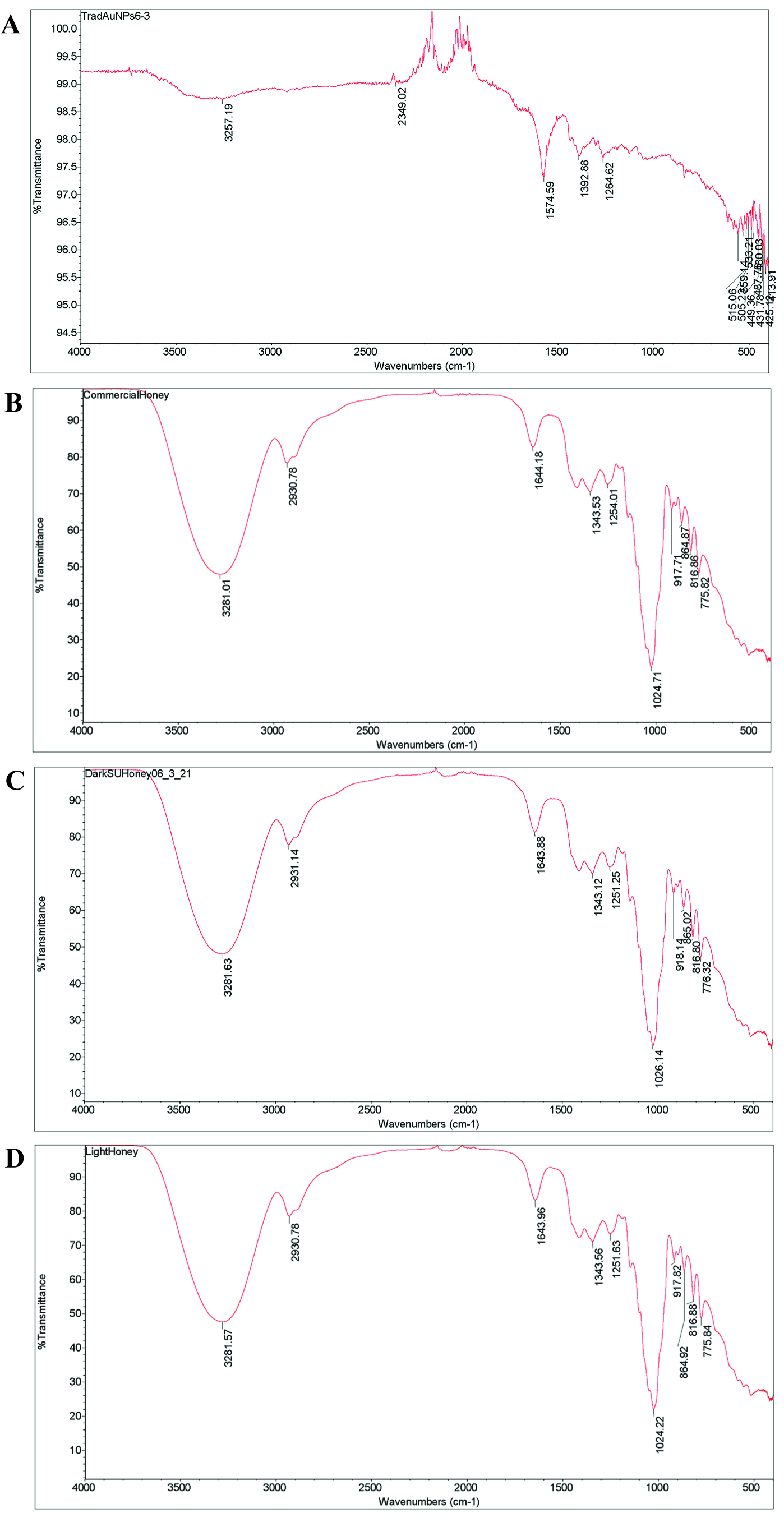

Supplement: S2 Data — (TIF) [file pone.0291076.s004.tif]
